# Prevalence of Cardiovascular Risk Factors Among Adults in the European Union: A Systematic Review with Meta-Analysis

**DOI:** 10.3390/jcm14165752

**Published:** 2025-08-14

**Authors:** Jennifer Sacramento-Pacheco, María Begoña Sánchez-Gómez, Gonzalo Duarte-Clíments, Juan Gómez-Salgado, María Mercedes Novo-Muñoz

**Affiliations:** 1 Nuestra Señora de Candelaria Nursing University School, University of La Laguna, 38010 San Cristóbal de La Laguna, Spain; 2 Europe South Training Center (Cesur Tenerife), 38006 Santa Cruz de Tenerife, Spain; 3 Cátedra de Enfermería, University of La Laguna, 38200 San Cristóbal de La Laguna, Spain; 4 Research in Health and Social Care Sciences Program, Catholic University of Murcia (UCAM), 30107 Murcia, Spain; 5 Cieza Este Health Centre, Murcia Health Service, 30530 Murcia, Spain; 6 Faculty of Health Sciences, International University of Valencia (VIU), 46002 Valencia, Spain; 7 Department of Sociology, Social Work and Public Health, Faculty of Labour Sciences, University of Huelva, 21007 Huelva, Spain; 8 Safety and Health Postgraduate Program, Universidad Espíritu Santo, Guayaquil 092301, Ecuador; 9 Research Group IENFERCAN, Department of Nursing, Faculty of Nursing, University of La Laguna, 38200 San Cristóbal de La Laguna, Spain

**Keywords:** heart disease risk factors, obesity, diabetes mellitus, prevalence, European Union, cardiovascular risk factors, public health

## Abstract

**Background**: Cardiovascular diseases are the leading cause of death in Europe. The cardiovascular risk factors (CVRFs) with the greatest clinical impact are high blood pressure (HBP), type 2 diabetes mellitus (DM2), smoking, overweight, obesity, dyslipidaemia, and sedentary lifestyle. The objective of this review was to compare the prevalence of the different CVRFs according to population-based studies conducted in the European Union. **Methods**: This systematic review aimed to assess the prevalence of CVRFs in the European Union. A comprehensive search strategy was employed, including databases such as PubMed, Scopus, and Web of Science, using specific keywords related to cardiovascular risk factors, prevalence, and European countries. The quality of the reviewed studies was assessed using established criteria, categorising them as low, moderate, or high quality. **Results**: A total of 26 studies from Belgium, Croatia, Cyprus, Czech Republic, Estonia, France, Germany, Greece, Hungary, Ireland, Italy, Lithuania, Luxembourg, the Netherlands, Poland, Portugal, Romania, Spain, and Sweden were included. The findings revealed considerable variability in the prevalence of CVRFs across the European Union, with overweight, hypercholesterolemia, and hypertension being the most frequently reported. Prevalence rates varied notably by country, age group, and sample characteristics. The majority of the included studies were of moderate methodological quality, underscoring the need for more rigorous research to better support evidence-based policies and targeted health interventions. **Conclusions**: There are differences between the studies presented, ranging from the sex ratio, the age studied, to even the CVRFs included. This also means that the prevalence of each CVRF varies by country.

## 1. Introduction

Cardiovascular diseases (CVDs) encompass a range of conditions affecting the heart and blood vessels, including cerebrovascular diseases like stroke, coronary artery disease, and peripheral artery disease [1]. In Europe, CVDs remain the leading cause of mortality, responsible for approximately 45% of all deaths across the continent. Among these, ischaemic heart disease stands as the most prevalent cause of death and a major contributor to overall CVD mortality in many European nations [2,3,4,5,6,7,8,9]. The highest mortality rates for ischaemic heart disease are observed in countries such as Slovakia, Lithuania, Latvia, and Hungary, while the lowest rates are recorded in France and the Netherlands [10]. Over the past quarter century, the incidence of CVDs in Europe has risen, leading to a significant decline in both quality of life and healthy life expectancy [5,11].

This increase in CVD cases imposes substantial financial burdens on healthcare systems, particularly in managing individuals with cardiovascular conditions or those at high risk. However, this burden could be alleviated through effective health promotion and disease prevention strategies, with nursing playing a central role in these efforts [1,9,11]. To assess cardiovascular risk, various stratification tools, including REGICOR, SCORE, and the Framingham Risk Score, are commonly used. The Framingham tool, the first of its kind, requires adaptation for different populations to ensure accuracy [1].

Cardiovascular risk factors (CVRFs) are characteristics that elevate the likelihood of developing CVDs. These factors are divided into modifiable and non-modifiable categories. Non-modifiable factors, such as age, sex, and ethnicity, cannot be altered, while modifiable factors, which are of greater clinical significance, can be managed through preventive measures. These include hypercholesterolemia, hypertension, diabetes mellitus (DM), smoking, obesity, overweight, sedentary behaviour, and conditions like metabolic syndrome and abdominal obesity [12]. Proper management of CVRFs, combined with a healthy lifestyle, can significantly reduce the risk of cardiovascular events [13]. Consequently, understanding the prevalence and trends of CVRFs is crucial for predicting the future progression of CVDs [14].

While significant attention has been focused on identifying CVRFs, there remains a critical gap in understanding how these factors evolve over time and their long-term effects on both individual health and healthcare systems. The variation in CVRF progression across different regions of the European Union further complicates the task of predicting CVD outcomes and the resources required for their management. Understanding how these disparities in risk factor development play out in different socio-economic, cultural, and healthcare contexts is crucial. Such knowledge is not only necessary for making informed policy decisions but also for designing localised, context-specific interventions that can effectively prevent cardiovascular diseases. Bridging this gap in knowledge will lead to more accurate predictions, enabling public health policies to be both more precise and regionally tailored, reducing the overall CVD burden in Europe while ensuring more equitable access to care.

The aim of this study was to compare the prevalence of CVRFs as reported in studies conducted across the European Union countries.

## 2. Materials and Methods

### 2.1. Protocol

This systematic review of the published literature on the prevalence of CVRFs in the European Union was conducted in accordance with the Preferred Reporting Items for Systematic Reviews and Meta-Analyses (PRISMA) guidelines [15,16]. To ensure transparency and methodological rigor, a completed 27-item PRISMA checklist has been submitted and all relevant items are addressed throughout the manuscript.

The research question, according to the format Condition, Context, Population (CoCoPop) [17] was as follows: What is the prevalence of cardiovascular risk factors in the population over 18 years of age in the European Union?

The protocol of the systematic review has been registered (https://doi.org/10.17605/OSF.IO/Z86XD) [18].

### 2.2. Eligibility Criteria

All publications examining the prevalence of cardiovascular risk factors (CVRFs) in the adult population of the European Union countries were considered for inclusion [19,20]. Studies were excluded if they did not estimate the prevalence of CVRFs, did not focus on adult population groups, assessed prevalence only in specific or clinically diagnosed subgroups, or presented results that were not applicable to the context of this review. Publications that were considered to lack sufficient methodological quality based on the critical evaluation tools were also excluded.

Additionally, certain study designs were excluded such as consensus documents, academic essays, letters to the editor, editorials, and pilot studies. No restrictions were applied to publication date or language in order to ensure comprehensive coverage of the available literature.

### 2.3. Information Sources

The electronic databases consulted included PubMed, the Virtual Health Library (VHL), SciELO, the Red de Revistas Científicas de Acceso Abierto Diamante (Redalyc), Dialnet, and the Latin American and Caribbean Health Sciences Literature (LILACS). The search was complemented by a manual review of references. The primary literature search was conducted through PubMed and Scopus, two major biomedical and health science databases. In this process, Scopus yielded a high degree of overlapping with PubMed, and duplicate records were excluded to streamline the review. Additional region-specific databases, such as SciELO and Redalyc, provided relevant studies that contributed to broader coverage.

### 2.4. Search Strategy

The databases were queried using the search equations detailed in Table A1. The search strategy was developed based on the study’s objectives, incorporating relevant keywords and their corresponding MeSH terms.

### 2.5. Selection of Studies

A pre-selection was carried out by reading titles and abstracts. Full-text references were obtained. In one case, it was necessary to contact the authors to retrieve the publication. Duplicates were removed, and articles were critically read in full and assessed using the CASPe tool for reviews [21] and for cohort studies [22]. The Berra et al. tool was used for cross-sectional studies [23], and synthesis of the evidence was also carried out using the Scottish Intercollegiate Guidelines Network tool (SING) [24]. This analysis is detailed in Table 1.

### 2.6. Data Collection

Ad hoc forms were devised for independent and peer-reviewed assessment of the main characteristics of each included study. Two reviewers independently conducted the data extraction to ensure accuracy and consistency. The data selected by the research team were extracted for the purpose of writing and synthesising the sections of the study in response to the stated objective. Due to variability in the reporting quality of the included studies, not all sources provided complete information on sample sizes or confidence intervals for prevalence estimates. As a result, the extracted data were presented in a simplified and harmonised format, focusing on prevalence ranges to ensure clarity and consistency across studies. This approach was taken to avoid introducing bias or inconsistencies stemming from selective reporting. When available, exact values and confidence intervals were retained, and they are presented in the Appendix A to enhance transparency.

These syntheses were compared in order to resolve any possible discrepancies between the reviewers. In cases of disagreement, issues were discussed in detail until consensus was reached and, when necessary, a third reviewer was consulted to provide an independent judgment and facilitate resolution.

### 2.7. Data Aggregation

The results obtained have been presented on the basis of the CVRFs reported in each study by the European Union country and expressed as a percentage of the total population included in each study, differentiated by sex (where available) and stating total prevalence.

### 2.8. Risk of Bias

The risk of bias was assessed after critical appraisal using the CASPe tool for reviews [21] (Critical Appraisal Skills Programme—Spanish adaptation), which evaluates the clarity of the research question, methodological rigour, precision, and applicability; a minimum score of 8/10 was required. For cohort studies, the CASPe checklist specific to cohort designs [22] was applied, with a minimum score of 11/11 for inclusion. In the case of cross-sectional studies, the Berra et al. instrument was used [23], appraising sampling strategy, measurement validity, control of confounding, and precision; all included studies were rated as high quality. Additionally, the Scottish Intercollegiate Guidelines Network (SIGN) framework was employed to grade the overall level of evidence and ensure consistency in judgments across study designs. All tools were piloted and applied independently by two reviewers, with minor wording adaptations to align with the epidemiological focus of our question; disagreements were resolved by consensus, or by consulting a third reviewer when necessary (Table 1).

### 2.9. Synthesis Methods

The synthesis of the evidence was conducted in accordance with the Scottish Intercollegiate Guidelines Network (SIGN) [24], resulting in a level of evidence (LE) of 2++ and a degree of recommendation (DR) of B according to the LE (Table 1).

### 2.10. Reporting the Risk of Bias

Critical appraisal and synthesis of the evidence were conducted to ensure that the included studies were without bias. This was carried out by incorporating a third reviewer into the synthesis process.

### 2.11. Assessment of Certainty

The certainty of critical appraisal was ensured by using the CASPe critical reading tool [21,22] and the Berra et al. tool [23]. In addition, a synthesis of the evidence was carried out using the Scottish Intercollegiate Guidelines Network (SIGN) [24] (Table 1).

Data from this assessment were collected in ad hoc, double-blind questionnaires in order to ensure the proposed certainty based on the available evidence.

### 2.12. Data Analysis

A comprehensive description of the primary features of the selected studies was conducted, along with a quantitative evaluation of the data aligned with the objectives of the review. The meta-analysis of prevalence data was carried out using Stats Direct software (version 3.0.0, Stats Direct Ltd., Cheshire, UK). To estimate the effect sizes and proportions in each study, 95% confidence intervals (CIs) were applied. The heterogeneity among studies was examined using Cochran’s Q test (significance threshold set at 10%) and quantified through the I^2^ statistic. An I^2^ value exceeding 50% was interpreted as evidence of substantial heterogeneity.

Given the variability detected across studies, a random-effects model—specifically, the DerSimonian and Laird method—was adopted instead of a fixed-effects approach to compute the pooled estimates, using a 95% CI. Forest plots were employed to visually represent the heterogeneity, illustrating individual study proportions alongside the overall prevalence. For aggregated results, both the pooled proportion and the corresponding 95% CI were reported. Finally, potential publication bias was assessed using Egger’s linear regression test, with *p*-values below 0.10 suggesting statistically significant bias. This threshold was selected in accordance with commonly accepted practice in meta-analyses, particularly because Egger’s test has low statistical power when the number of included studies is small, making a more conservative cut-off (e.g., *p* < 0.05) potentially less sensitive to detect true asymmetry.

## 3. Results

### 3.1. Review of Main Results

**Figure 1:**
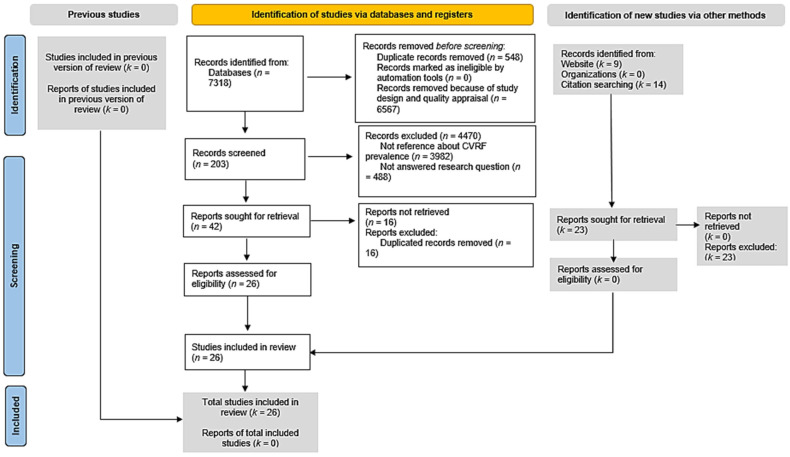
PRISMA 2020 flow diagram.

Figure 1 shows the selection process of the 26 identified studies [2,4,9,25,26,27,28,29,30,31,32,33,34,35,36,37,38,39,40,41,42,43,44,45,46,47]. The studies included observational cross-sectional studies and two reviews of the literature.

Results were obtained from countries belonging to the European Union: Belgium, Croatia, Cyprus, Czech Republic, Estonia, France, Germany, Greece, Hungary, Ireland, Italy, Lithuania, Luxembourg, Netherlands, Poland, Portugal, Romania, Spain, and Sweden. The first study (EUROASPIRE) estimated overall European prevalence of CVRFs (Table 2).

The results obtained from studies conducted in various European Union countries reveal notable differences in the prevalence of CVRFs, both between countries and among regions within them. These variations appear to be associated with disparities in observed cardiovascular outcomes.

EUROASPIRE V, a European survey carried out in 27 countries with 8261 participants, found that—with the exception of smoking—all CVRFs were more prevalent among women [9]. However, this trend is reversed in several national studies, where CVRFs are more prevalent among men [26,28,30]. Other studies show a more balanced or variable distribution depending on the specific risk factor [4,27,29,32,34,35,38,40,43,44,45].

In Germany, for instance, pronounced regional differences were observed: arterial hypertension was most prevalent in Saxony (39.4% among women) and was least prevalent in Bremen (27.4%) [4]. In contrast, in Italy, hypertension reached its highest prevalence in the northeast (73.4% among men) and reached its lowest prevalence in the islands (61%) [35]. Similar territorial disparities were seen in Spain, where hypertension ranged from 22% in Castilla and León to 45% in Barcelona, and diabetes mellitus (DM) ranged from 10% (Madrid/Navarra) to 22% (Barcelona) [43,46].

Other countries exhibit unique characteristics: In Croatia, a high prevalence of CVRFs was reported among the Roma population compared to the general population [26]; in Portugal, Portuguese emigrants in Switzerland showed different prevalence rates compared to residents in Porto [41]; and in Poland, studies revealed significant differences between northern and southern regions, as well as between urban and rural areas [39,40].

The MONICA, MONALISA, and ELISABET studies highlighted the temporal evolution of CVRFs from 1986 to 2007, with persistent rates of obesity and dyslipidaemia [31]. Similarly, in Greece, a review of 22 population-based studies conducted between 1994 and 2016 demonstrated significant variability over time and across studies, reflecting a progressive epidemiological transition [2].

Age was also a key factor in many studies. In Cyprus, research on individuals over 65 years old revealed a relationship between cardiovascular health and depression [27], while in Ireland, studies focused on adults aged 55 to 67 [33]. In Sweden, cohorts born between 1943 and 1953 showed generational differences in the prevalence of CVRFs [47].

Overall, the most frequently assessed CVRF was hypertension, followed by smoking and dyslipidaemia, although some studies did not include factors such as overweight, obesity, or diabetes [25,30,32,36,39]. Moreover, the definition and number of CVRFs assessed varied considerably across studies, complicating direct comparisons.

In summary, differences in the prevalence of CVRFs both between and within countries appear to correlate with disparities in cardiovascular outcomes. Regions with higher prevalence of hypertension, obesity, or diabetes tend to exhibit worse cardiovascular profiles, underscoring the need for prevention strategies tailored to the specific sociodemographic and geographic context of each country.

### 3.2. Individual Study Results and Characteristics

#### 3.2.1. EUROASPIRE V

EUROASPIRE V was a survey conducted between 2016 and 2017 in 27 countries. It included a total of 8261 participants, of whom 74.3% (6132) were men and 25.7% (2129) were women between 18 and 80 years of age [9].

The assessed CVRFs and the total prevalence by sex and age group are shown in Table 3.

#### 3.2.2. Belgium

A study was conducted involving 1017 participants, of which 50.1% were women and 49.9% were men. The study was conducted in the city of Liège, Wallonia [25]. Table 3 shows the CVRFs included and the prevalence obtained. For all of them, the total prevalence and the prevalence by age group are presented for each sex: 20 to 29 years (G1), 30 to 49 years (G2), and 50 to 69 years (G3). The age group with the greatest representation was G2.

#### 3.2.3. Croatia

In 2013, a study was published in which the prevalence of CVRFs in a Croatian Roma population group was assessed and compared with nationally published data [26]. The included CVRFs are listed in Table 3.

The Croatian sample (with which the study was compared) comprised 19,070 individuals between 18 and 65 years of age. The percentage of each sex is unknown. Total prevalence of each CVRF by sex are shown in Table 3, but the total prevalence is not shown.

The Roma population had an initial sample of 430 participants aged between 18 and 84 years, where 35.12% were men (151) and 64.88% were women (279). The total prevalence rates of the sample and the prevalence by sex are presented in Table 3.

#### 3.2.4. Cyprus

A study was conducted to examine the relationship between CVRFs and depression in older adults. The sample size was 300 participants over 65 years of age, where 45.34% were men (136) and 54.66% were women (164) [27].

The study presents the prevalence of CVRFs by sex and estimates total prevalence, as detailed in Table 3.

#### 3.2.5. Czech Republic

A study was carried out between 2014 and 2016. A sample of 1051 individuals was selected, of which 45.86% were men (482) and 54.14% were women (569). The age range was not specified, although it is known that the mean age was 45.6 ± 14.7 in men and 44.8 ± 13.7 in women [28].

Another study was conducted in 2020. The sample consisted of 1812 participants. Men were over 40 years of age, accounting for 53.03% (961), and women were over 50 years of age, accounting for 46.97% (851) [29].

CVRFs and their prevalence are shown in Table 3. Based on the prevalence by sex and the number of participants of each sex in each study, the total prevalence of the sample studied can be estimated [28,29].

#### 3.2.6. Estonia

In Tallinn, a study was conducted with *N* = 1111 participants, aged 20–65 years, of whom 46% were men (511) and 54% were women (600) [30].

The prevalence of CVRFs by sex and total prevalence are presented in Table 3.

Cardiovascular risk (the Framingham scale) was calculated, with a >10% risk of coronary heart disease found in 34.6% of men and 14.4% of women and a total prevalence of 23.7% [30].

#### 3.2.7. France

A study was conducted aiming to compare the prevalence of CVRFs in different years including samples from previous studies. From the MONICA 1986–1988 study, a sample of 860 was obtained; in MONICA 1995–1996, the sample was 1021; it was also 1021 in MONALISA 2005–2007; in the ELISABET study, the sample was 1636. All participants were aged between 40 and 64 years of age [31].

For each sample and study, total prevalence by sex is presented, allowing for the estimation of total prevalence. Prevalence data and the CVRFs included are presented in Table 3.

#### 3.2.8. Germany

The GEDA study was conducted between 2009 and 2012 and included men and women aged 18–99 and 18–100, respectively. A total of 28,033 men and 34,573 women participated. This amounts to a percentage of 48.9% males and 50.9% females. Participants from different German federal states were included, with higher participation from North Rhine-Westphalia and lower participation from Bremen (for both sexes) [4].

The prevalence of the CVRFs is presented in Table 3 for each of the states included and by sex.

Regional differences were observed across the different participating federal states. Among women, sedentary lifestyle had the highest prevalence in Mecklenburg-Pomerania (41.2%) and the lowest in Baden-Württemberg (31%); risky alcohol use had the highest prevalence in Hamburg (24.6%) and the lowest prevalence in Mecklenburg-Pomerania (16.6%); smoking had the highest prevalence in Bremen (30.9%) and the lowest prevalence in Saxony (20%); obesity was most prevalent in Saxony-Anhalt (23.2%) and least in Bremen (10.4%); HBP was most prevalent in Saxony (39.4%) and least in Bremen (27.4%); diabetes was most prevalent in Saxony-Anhalt (13.6%) and least in Baden-Wurttemberg (7.2%); the prevalence of dyslipidaemia was highest in Saarland (34.8%) and lowest in Saxony-Anhalt (23.2%); finally, the prevalence of low fruit and vegetable intake was highest in Saarland (14.5%) and lowest in Saxony (8.3%). In the case of men, sedentary lifestyle was most prevalent in Saxony-Anhalt (44.1%) and lowest in Baden-Württemberg (31.7%); risky alcohol use was highest in Saxony (40.1%) and lowest in Bremen (26%); smoking was most prevalent in Berlin (40.7%) and least in Saxony (28.3%); obesity was most prevalent in Mecklenburg-Pomerania (20%) and least prevalent in Hamburg (10.4%); HBP prevalence was highest in Saxony-Anhalt (42.5%) and lowest in Hamburg (23.9%); diabetes prevalence was highest in Brandenburg (13.4%) and lowest in Bremen (3.6%); prevalence of dyslipidaemia was highest in Rhineland-Palatinate (34.7%) and lowest in Schleswig-Holstein (27.1%); prevalence of low fruit and vegetable intake was highest in Saarland (28.1%) and lowest in Saxony (15.4%) [4].

#### 3.2.9. Greece

In 2019, a literature review was published examining the prevalence of CVRFs according to different population-based studies [2].

It includes a total of 22 population-based studies and national health surveys for which data were collected from 1994 to 2016, allowing for the visualisation of the evolution of the CVRFs exposed. The total prevalence data of the sample are presented, including differences across studies [2].

In chronological order, the studies included ranged from 1994 to 2015. The prevalence rates obtained are detailed in Table 3.

#### 3.2.10. Hungary

A study was conducted in 2008 involving 546 volunteers aged 14–83 years. The results of the CVRFs are presented in Table 3. Percentage data for men and women are not available, so total prevalence cannot be calculated [32].

#### 3.2.11. Ireland

Using data from the Irish longitudinal survey, the overall prevalence of HBP, DM, sedentary lifestyle, smoking, hypercholesterolaemia, and overweight/obesity were studied in a population-based sample following a cross-sectional study [33]. This study included 4330 participants with no previous diagnosed cardiac pathology, of whom 44.5% were men and 55.5% were women, with an age range between 55 and 67 years. The results are detailed in Table 3.

#### 3.2.12. Italy

The ‘BORDERLINE’ study assessed the prevalence of CVRFs in Italy, and reported the total results and by sex. The total sample was 692 participants, 54.5% (377) women and 45.5% men (315). The participants had a mean age of 60 with an SD of 13.2 [34].

In the same year, the results of a national cross-sectional survey were also published, involving 24,213 volunteers over 18 years of age, of whom 52% were men (12,626) and 48% were women (11,587). The study participants belonged to different Italian regions. Thus, total prevalence by sex and also by region of Italy are presented. Given that the total prevalence by sex is presented and the percentage of participants of each sex is known, the total prevalence of CVRFs can be calculated [35].

The study shows the prevalence of CVRFs by regions of Italy, divided into north-west, north-east, south, centre, and islands. The prevalence of HBP for both sexes was highest in the north-west and north-east (73.4% of men in both regions and 52.1% of women only in the north-west) and lowest in the islands (61% of men and 40.6% of women); the highest prevalence of DM was found in the south for both sexes (21.5% and 12.6% of men and women, respectively) and the lowest prevalence was in the centre (16.8% of men and 9.6% of women); the highest prevalence of obesity was found in the south (31.5% of men and 23.5% of women) and the lowest prevalence was in the north-east (19.9% of men and 12.9% of women); the highest prevalence of overweight corresponded to the centre and islands for men (57.5%) and the centre for women (32.5%), and the lowest prevalence was found in the south for men (52.6%) and in the north-west for women (29.2%); the highest prevalence of abdominal obesity was found in the islands for men (61.5%) and in the south for women (60.6%); the highest prevalence of hypercholesterolaemia in men and women was in the north-east (29.7% and 35.2%, respectively) and the lowest prevalence was in the islands for men (24.6%) and in the south for women (29.3%); the highest prevalence of hypertriglyceridaemia was reported in the north-east for men (52%) and in the north-west for women (48.1%) and the lowest prevalence was in the islands for both sexes (43.1% of men and 36.5% of women); the prevalence of dyslipidaemia was highest in the north-east for men (65.2%) and north-west for women (63.3%) and the lowest in the islands for men (56.2%) and in the south for women (52.2%); smoking had a higher prevalence in the islands for both sexes (30.5% of men and 17.9% of women) and a lower prevalence in the north-west (18.8% of men and 11.6% of women) [35].

The prevalence results by sex and totals are shown in Table 3.

#### 3.2.13. Lithuania

A study was conducted involving 435 volunteers aged between 40 and 60 years from six geographical areas of the country: western, eastern, and southern high and low Lithuania [36]. Of the total number of volunteers, 49.7% (216) were men and 50.3% (219) were women. The total prevalence of the sample is presented in Table 3.

#### 3.2.14. Luxembourg

The ORISCAV-LUX study involved 1361 participants aged 18–69 years. The prevalence of CVRFs in the participants diagnosed with chronic kidney disease (CKD) and those not diagnosed with CKD is presented. Similarly to the other studies, the total prevalence of the sample without CKD is shown in Table 3. The sample of participants without CKD was 1270 participants, 48.7% (618) of whom were men and 51.3% (652) were women. The prevalence of DM in the sample was found for 1331 participants [37].

#### 3.2.15. The Netherlands

In 2016, a study was published in which the prevalence of CVRFs in the Netherlands was compared with that in China. The Dutch sample was drawn from the Utrecht Health Project (UHP) [38] study cohort and comprised a total of 6542 participants (44.8% men and 55.2% women) aged 18 years and older. Prevalence rates are presented by sex and age group. Based on the percentage of participants of each sex and the prevalence of each CVRF, the total prevalence can be estimated. The percentages are detailed in Table 3.

#### 3.2.16. Poland

Trzeciak et al. conducted a study comparing the prevalence of CVRFs in the northern and southern Polish population [39].

The sample consisted of 7376 participants, of whom 60.9% (4495) were from the north of Poland and 39.1% (2881) were from the south, aged between 40 and 65 years [39]. Total prevalence rates can be estimated by looking at the prevalence between north and south and by knowing the number of individuals in each area.

The LIPIDOGRAM 2015 study conducted in Poland highlights the prevalence of CVRFs in a sample of 13,724 participants, of which 63.3% were women and 36.7% were men. In both sexes, there was higher participation in urban areas. The mean age was 56 in rural areas and 57 in urban areas, with an SD of 14 in both areas. In women, the mean age was 55 and 57 in rural and urban areas respectively, with an SD of 14; in men, the mean age was 56, with an SD of 14 [40].

Table 3 shows the prevalence of CVRFs as total prevalence of the sample by sex and by rural/urban area.

#### 3.2.17. Portugal

A study was conducted in Porto (Portuguese residents) and Lausanne (Portuguese emigrants in Switzerland) [41].

The Porto sample consisted of 1550 individuals aged 35–65 years, of whom 37.3% (578) were men and 62.7% (972) were women. Table 3 shows the total prevalence of CVRFs in the sample.

#### 3.2.18. Romania

In 2023, a study was published in Romania showing the prevalence of CVRFs in a sample of 117 participants aged 30–79 years, with a representation of 54.7% of women and 45.3% of men [42].

Table 3 presents the prevalence of CVRFs longitudinally according to the data obtained at baseline, at 6 months, and at 12 months.

#### 3.2.19. Spain

The DARIOS study (a pooled study of different population-based studies in different autonomous regions) presents prevalence rates according to the different studies and as total prevalence. The Spanish autonomous regions included are Andalusia, Balearic Islands, Canary Islands, Castilla-La Mancha, Castilla and Leon, Catalonia, Extremadura, Madrid, Murcia, and Navarre.

The study included 27,903 participants aged 35–74 years, where 54% were women and 46% were men. The highest prevalence rates were obtained in the ARTPER study (Barcelona), with a prevalence of HBP at 45%, DM at 22%, and dyslipidaemia at 57%. The lowest prevalence rates were obtained in Castilla and Leon for HBP and dyslipidaemia, with a prevalence of 22% and 25%, respectively, and for DM, with a prevalence of 10%, in Madrid, Castilla and Leon, and Navarre [43].

In a subsequent study, the prevalence of obesity and overweight by sex was published, and the total prevalence of the sample can be estimated. In this case, the population sample was 28,887 participants, of whom 53.5% were women and 46.5% were men in the same age group [44]. The prevalence of MS was also studied, in this case with a sample of 24,670 individuals [45].

Another systematic review comparing all the studies presented on the prevalence of CVRFs in Spain showed that the prevalence of CVRFs in Spain varied substantially depending on the autonomous region studied. A higher prevalence of HBP was found in one of the studies in Barcelona and a lower prevalence was observed in Murcia; DM was higher in Andalusia and lower in the Di@bet.es study at national level; a higher prevalence of hypercholesterolaemia was found in Andalusia and a lower prevalence was found in one of the studies in Barcelona; the prevalence of metabolic syndrome was higher in the Di@bet.es study at national level and lower in Navarre; obesity and overweight had a higher prevalence in Andalusia and Barcelona and a lower prevalence in Madrid; sedentary lifestyle was higher in Madrid and lower in Toledo; smoking was less prevalent in Barcelona, but in different studies; alcohol use was more prevalent in Extremadura and lower in Toledo; dyslipidaemia was more prevalent in Toledo and less prevalent in the Di@bet.es study at the national level; and finally, the prevalence of hypertriglyceridaemia was higher in Barcelona and lower in Navarra [46].

All results are shown in Table 3.

#### 3.2.20. Sweden

In Sweden, a study was conducted including a sample of men and women born in 1953 (*n* = 595 men and *n* = 667 women) and a sample of men born in 1943 (*n* = 665). This makes a total sample of 1927. Men born in 1953 accounted for 30.9% of the sample, women represented 34.6%, and men born in 1943 made up 34.5% of the sample [47]. The prevalence rates of CVRFs for each cohort are detailed in Table 3.

### 3.3. Risk of Bias

Each of the studies was critically appraised [21,22,23] and a synthesis of the evidence was carried out [24] (Table 1). The results shown for CVRFs for the age ranges studied could represent a bias given that many CVRFs increase with age. Despite this, the studies with this variation have been included as the results show similarities.

### 3.4. Synthesis of Results and Meta-Analysis

The critical appraisal and synthesis of the evidence are provided in Table 1.

A meta-analysis was conducted to estimate the prevalence of high blood pressure (HBP), diabetes mellitus (DM), obesity, overweight, and smoking, based on studies that provided the necessary data. All meta-analyses included studies conducted in populations over 18 years of age within the European Union (*n* = 46).

**Figure A1:**
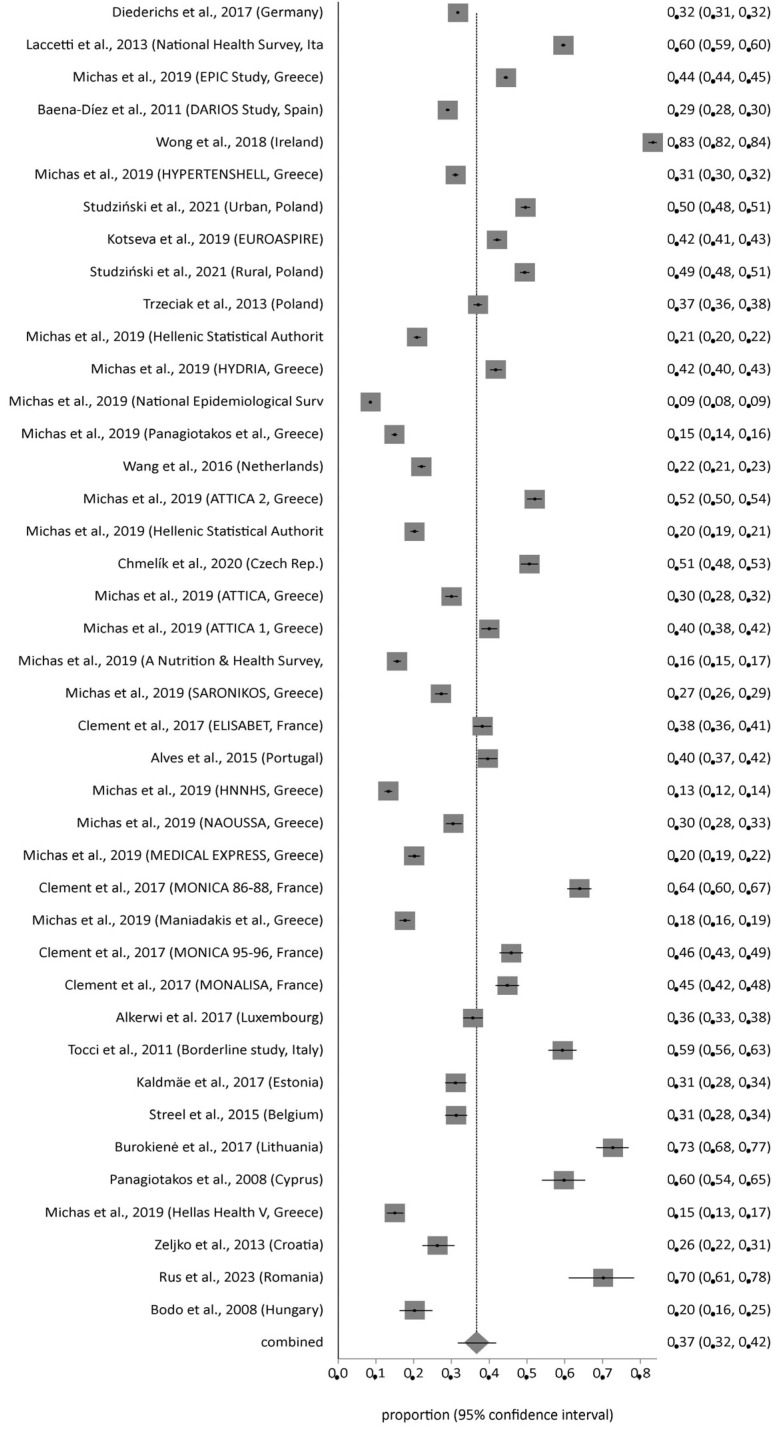
The forest plot for HBP [2,4,9,25,26,27,29,30,31,32,33,34,35,36,37,38,39,40,41,42,43].

**Figure A2:**
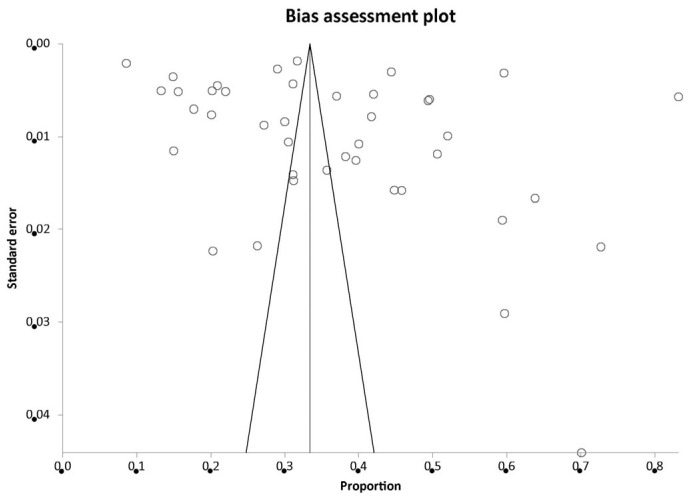
The funnel plot for HBP.

First, a meta-analysis on the prevalence of HBP was performed using the relevant studies. The total pooled sample included 96,609 individuals, yielding a prevalence of 36.64% (95% CI: 31.59–41.84%). The corresponding forest plot and funnel plot are presented in Figure A1 and Figure A2, respectively. The heterogeneity among studies was high, with an I^2^ value of 99.9%, while Egger’s test indicated no evidence of publication bias.

**Figure A3:**
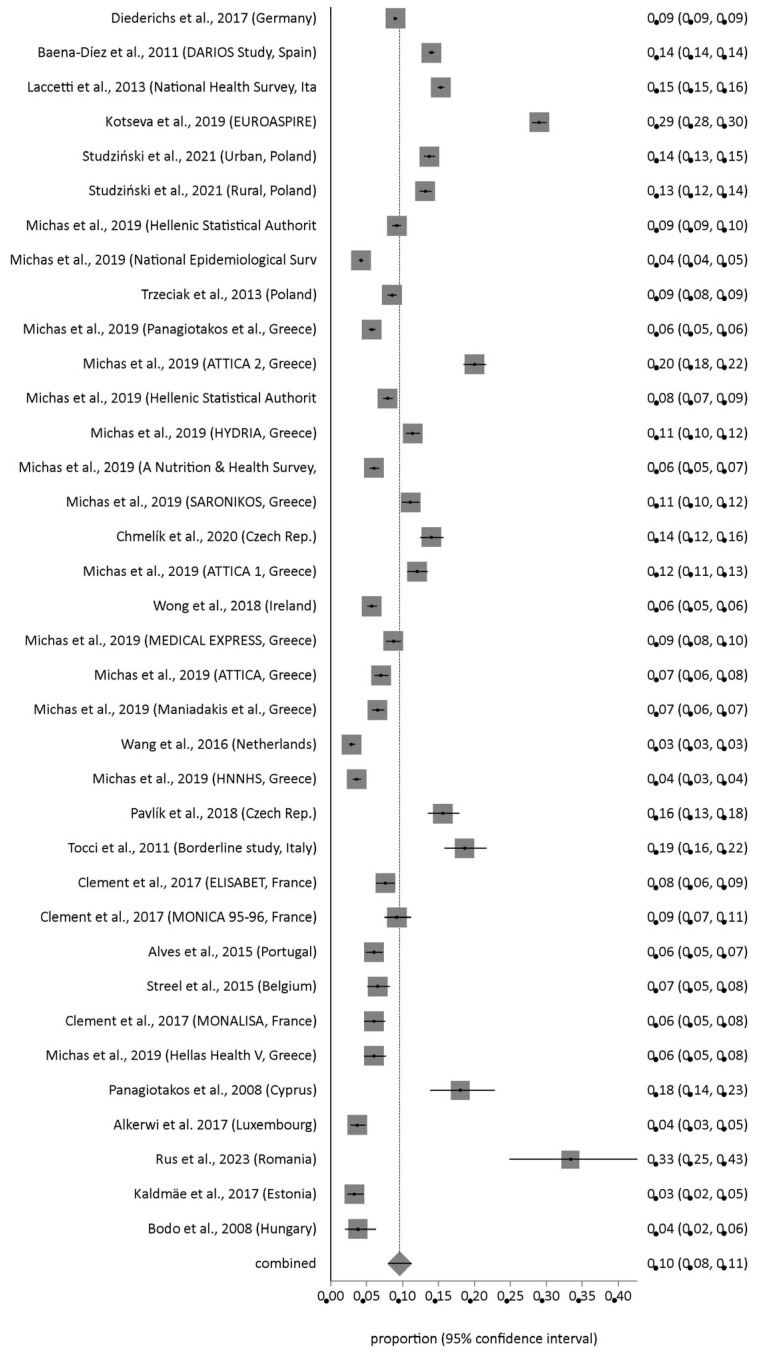
The forest plot for DM [2,4,9,25,27,28,29,30,31,32,33,34,35,37,38,39,40,41,42,43].

**Figure A4:**
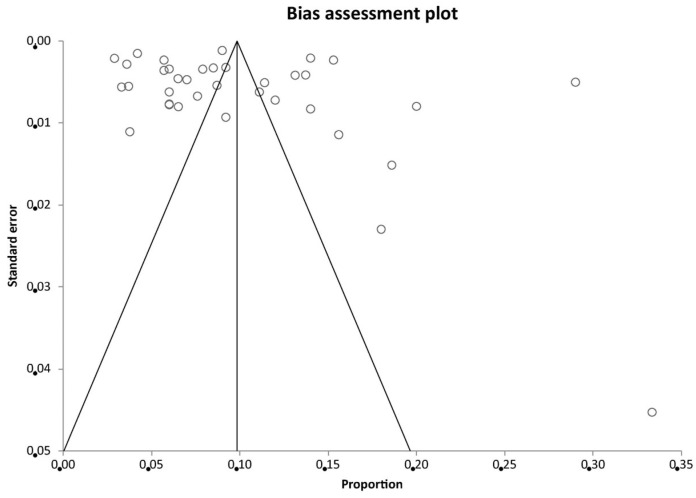
The funnel plot for DM.

On the other hand, a meta-analysis on the prevalence of diabetes mellitus (DM) was conducted across 36 studies. The total sample included in this analysis comprised 25,005 individuals with DM, yielding a pooled prevalence of 9.57% (95% CI: 7.96–11.32%). The corresponding forest plot and funnel plot are presented in Figure A3 and Figure A4, respectively. Heterogeneity was high (I^2^ = 99.5%), and Egger’s test indicated no evidence of publication bias.

**Figure A5:**
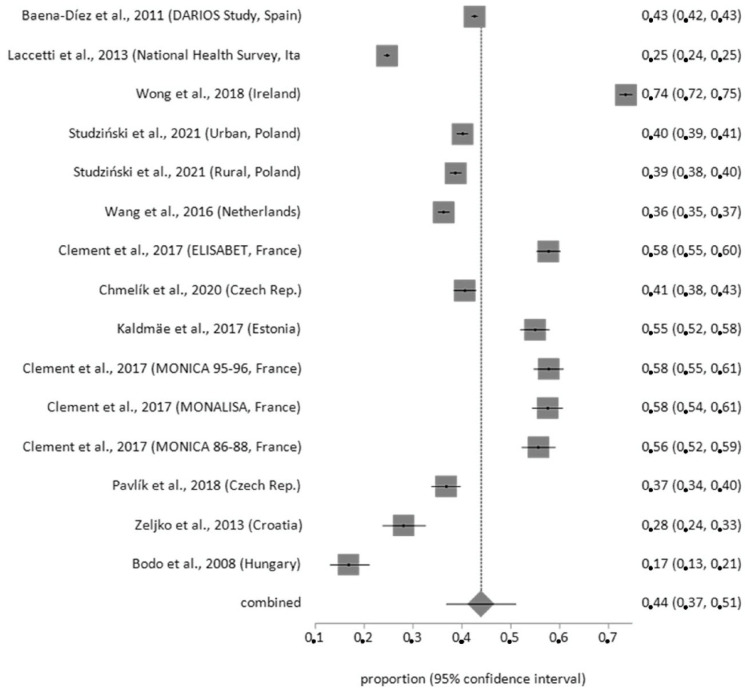
The forest plot for overweight [26,28,29,30,31,32,33,35,38,40,43].

**Figure A6:**
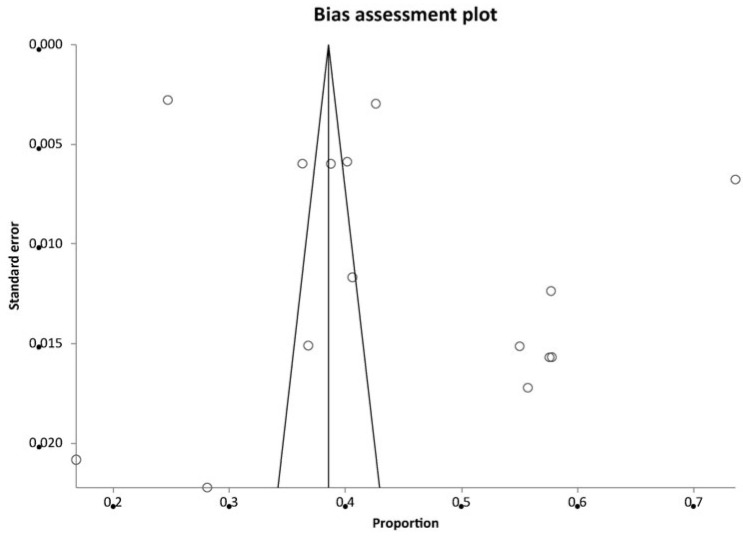
The funnel plot for overweight.

Regarding overweight, a prevalence meta-analysis was performed using data from 15 studies. The combined sample size was 33,361 individuals, with a pooled prevalence of 43.92% (95% CI: 36.83–51.12%). The forest plot and funnel plot are shown in Figure A5 and Figure A6, respectively. Heterogeneity was considerable (I^2^ = 99.7%), and Egger’s test showed no publication bias.

**Figure A7:**
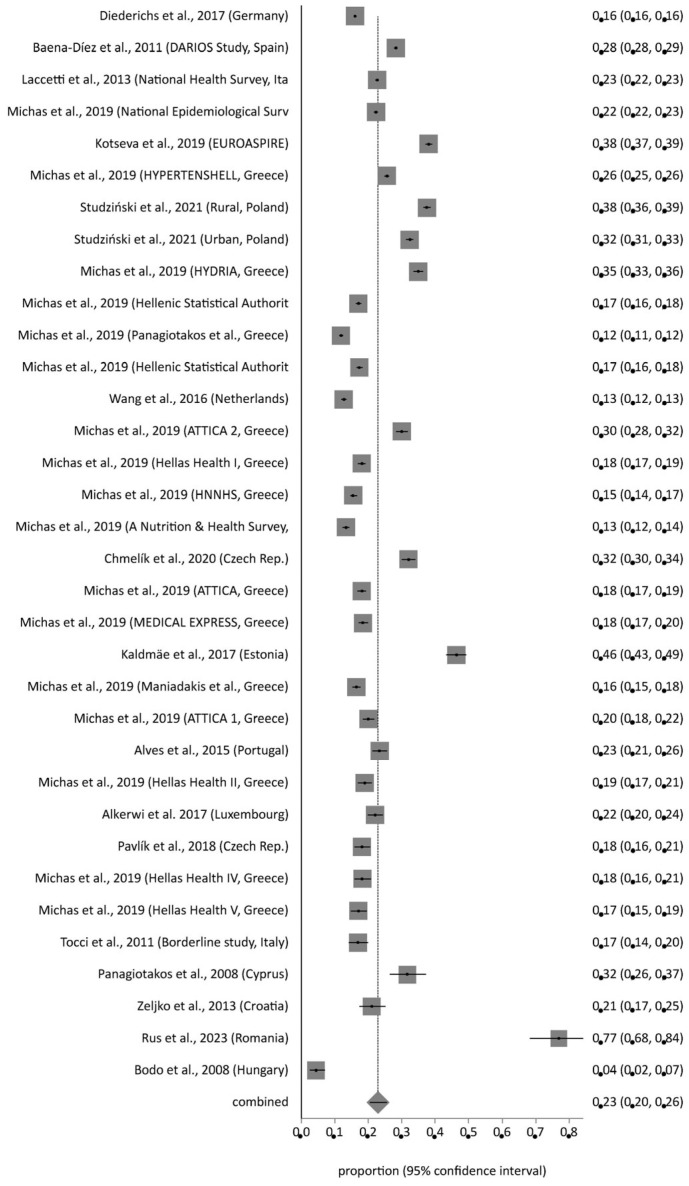
The forest plot for obesity [2,4,9,26,27,28,29,30,32,34,35,37,38,40,41,42,43].

**Figure A8:**
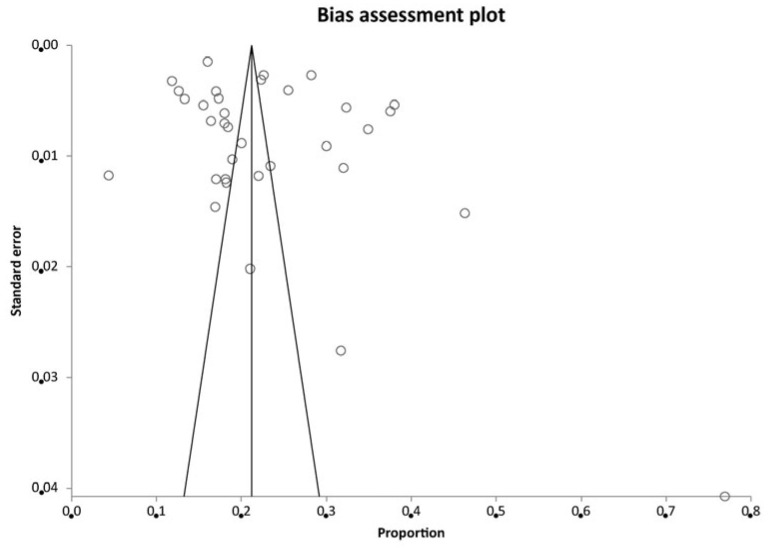
The funnel plot for obesity.

A separate meta-analysis on the prevalence of obesity included 34 studies with sufficient data. The total sample consisted of 57,419 individuals, and the overall prevalence was 22.89% (95% CI: 20.30–25.57%). The forest plot and funnel plot are displayed in Figure A7 and Figure A8, respectively. Heterogeneity remained high (I^2^ = 99.5%), with no publication bias detected according to Egger’s test.

**Figure A9:**
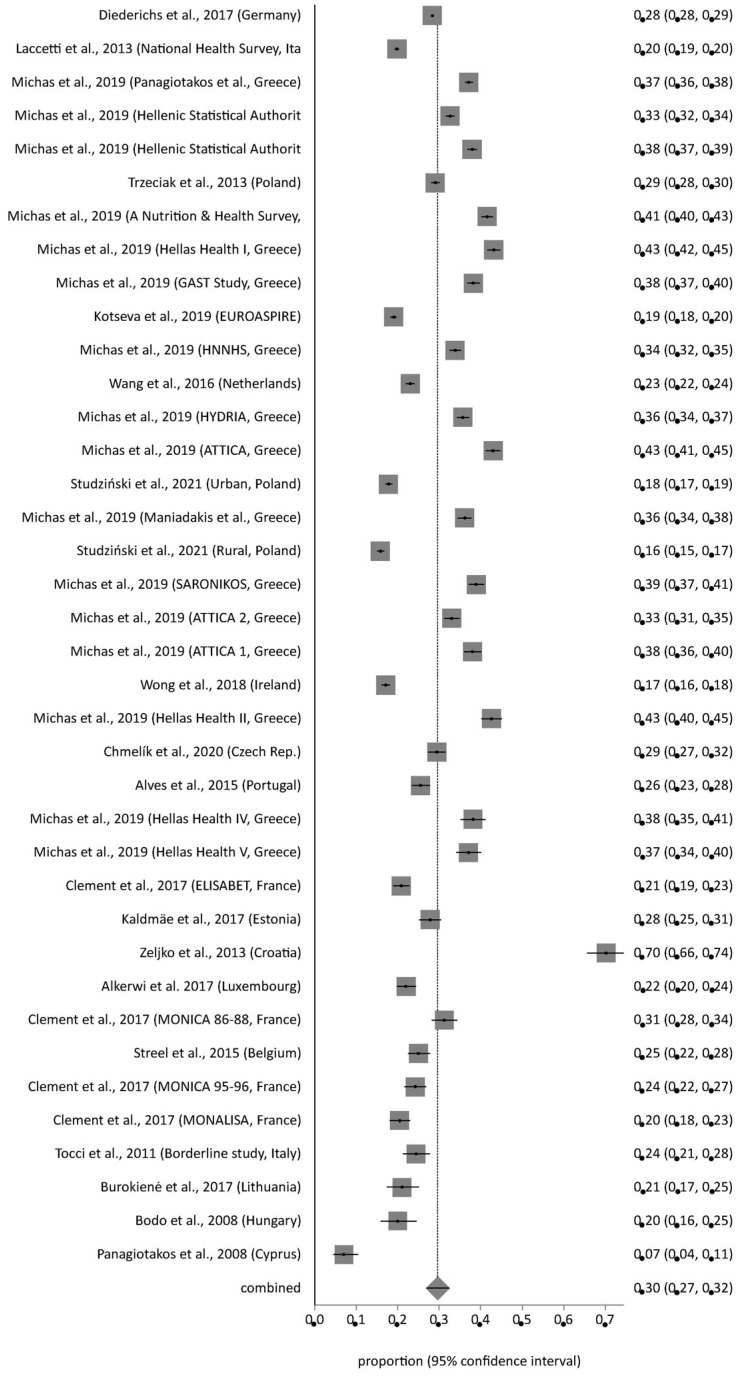
The forest plot for smoking [2,4,9,25,26,27,29,30,31,32,33,34,35,36,37,38,39,40,41].

**Figure A10:**
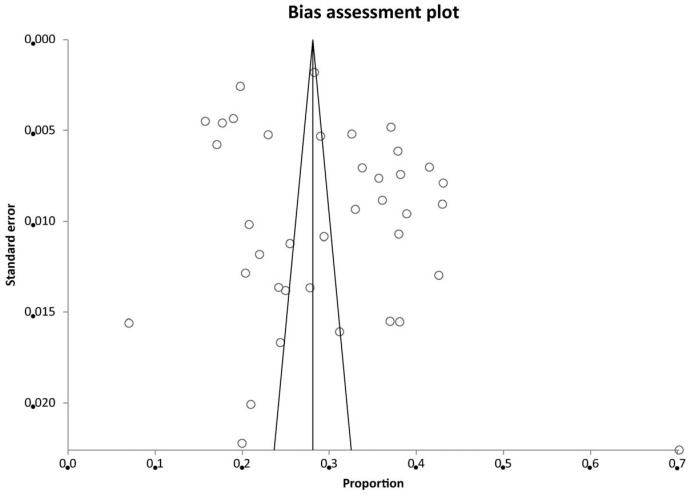
The funnel plot for smoking.

Finally, a meta-analysis of smoking prevalence was conducted using data from 38 studies. The total sample included 52,153 individuals, and the pooled prevalence was 29.65% (95% CI: 26.99–32.38%). The forest plot and funnel plot are provided in Figure A9 and Figure A10, respectively. The I^2^ value was 99.4%, indicating high heterogeneity, and no publication bias was observed based on Egger’s test.

## 4. Discussion

### 4.1. Interpretation of Studies

**Figure 2:**
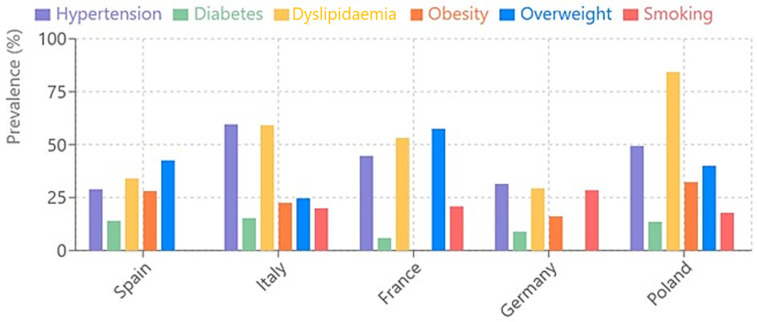
Prevalence of cardiovascular risk factors by country.

To create an effective visual representation, the most commonly reported cardiovascular risk factors across countries were selected. Only countries with sufficient data to allow for meaningful comparison were included. A grouped bar chart was used to facilitate a clear comparison of these risk factors by country (Figure 2).

A comparative bar graph showing the main cardiovascular risk factors was created for five European countries with more complete data: Spain, Italy, France, Germany, and Poland.

- It can be seen that Poland has the highest prevalence of dyslipidaemia (84.22%), which is significantly higher than in the other countries.
- Italy has the highest prevalence of hypertension (59.6%), followed by Poland (49.54%) and France (44.8%).
- France has the lowest value for diabetes (6%), while Italy has the highest value (15.3%).
- Overweight is particularly high in France (57.5%) and Spain (42.6%).
- Obesity varies considerably, being highest in Poland (32.34%) and lowest in Germany (16%).

The prevalence of CVRFs varies across studies. In four of the countries examined, there is a higher prevalence of the included CVRFs in males [26,28,30]; in the rest of the countries, variations exist across the two sexes [4,9,27,29,32,34,35,38,40,43,44,45]. In countries where CVRFs differ by sex, such differences are only found in one or two of the CVRFs surveyed, which have a higher prevalence in women, whereas all the CVRFs have a higher prevalence in men. In the case of the European survey, all CVRFs are more prevalent in women except smoking.

Differences exist between the CVRFs studied across studies. HBP is the most reported CVRF (not included in the study by Pavlík et al. [28]); smoking is addressed in all but two of the studies [28,42,43,44,45]; diabetes is not included in three studies [26,31,36]; overweight is excluded in nine studies [2,4,25,27,34,36,37,39,41]; obesity is not assessed in five countries [25,30,32,36,39]. It is worth noting that in the studies conducted in France [31] and Ireland [33], prevalence rates of overweight or obesity are presented together; hypercholesterolemia is not studied in 8 countries [4,25,29,31,36,37,40,43,44,45,46]. The remaining CVRFs appear in only a low proportion of studies, with dyslipidaemia (studied in only 6 countries) being the most common one [4,25,31,35,40,43,44,45,46]; hypertriglyceridaemia is only examined in 6 countries [26,28,30,32,35,46]; alcohol use is assessed in 6 countries [4,27,32,36,37,38]; sedentary lifestyle is present at the European level and is assessed in 6 countries [4,9,25,27,33,36,47]; and MS is studied in 3 countries [30,43,44,45,46].

The age range varies widely across studies, some with a wide age distribution with no maximum age range specified and others with a narrower age range [2,4,9,25,26,27,29,30,31,32,33,36,37,38,41,42,43,44,45,46]. There are studies in which the age range is not specified, but the mean age is available along with the standard deviation (SD) [28,35,40,47].

In those studies where data on the proportion of each sex in the sample are available, there is a higher representation of females in a greater number of studies than males. Only in the Czech Republic, France (MONICA 86–88 and MONALISA), and Italy studies is there a higher proportion of men [4,25,26,27,28,29,30,31,33,34,35,36,37,38].

According to data on the prevalence of CVRFs, in studies where the percentage of each sex is shown, dyslipidaemia, smoking, alcohol consumption, hypertriglyceridaemia, low intake of fruit and vegetables, and MS have a higher prevalence in males. HBP is a CVRF with higher prevalence in men except in Poland, Hungary, and the Croatian Roma population; the same is true for DM and overweight (except in Germany and Hungary); obesity has a higher prevalence in men, except in the Croatian Roma population, Spain, and the Italian BORDERLINE study; hypercholesterolaemia is more prevalent in men, except in the Croatian Roma population, Cyprus, Hungary, the Italian National Health Survey, and Poland.

Among men, the most prevalent CVRFs in decreasing order are overweight, hypercholesterolaemia, HBP, dyslipidaemia, sedentary lifestyle, and smoking; among women these are overweight, hypercholesterolaemia, HBP, sedentary lifestyle, dyslipidaemia, and smoking. The least prevalent, for both sexes, are DM, obesity, and smoking.

Certain circumstances in some studies should be highlighted. In France, four studies report a longitudinal observation of the evolution of CVRFs, such as HBP or dyslipidaemia, which show a decrease in prevalence, whereas DM and smoking experience a decrease followed by an increase. The opposite is true for the prevalence of overweight and obesity, which increase in prevalence in both sexes, although in the last study this prevalence decreases in women [31].

In Croatia, prevalence rates are presented for different populations, a Croatian sample and a Roma sample residing in Croatia [26].

In Greece, over the last 20 years, a number of population-based studies and health surveys have been conducted that provide data on the prevalence of CVRFs. Yet, not all studies report the same CVRFs. The study by Michas G et al. is a review of the literature which, given the interest in reflecting the prevalence rates obtained in different studies in the country, has been included in spite of not being a systematic review [2]. The variation in CVRFs is evident according to the follow-up of the ATTICA cohort and the ELSAT study. The prevalence of CVRFs has been increasing, with the exception of smoking. According to Hellas Health, the prevalence of obesity has remained stable while that of smoking has decreased [2].

In the study at Hungary, although the initial sample was 546 participants, for the exposed CVRFs, the final sample was 532, with the exception of hypercholesterolaemia and hypertriglyceridaemia, which was reported for 200 and 210 participants, respectively. Prevalence rates were calculated on the basis of this sample [32].

In Poland, total prevalence data and prevalence data by sex are presented, but with differentiation between rural and urban areas. Total prevalence rates for HBP, DM, overweight, and smoking are slightly higher in urban areas and lower in the case of dyslipidaemia, obesity, and abdominal obesity. When differentiated by sex, HBP, DM, and smoking have a higher prevalence in women in urban areas, and in the case of men this occurs with DM and overweight. The remaining factors have a lower prevalence in urban areas in both sexes, with HBP standing out in men, yet with almost equal prevalence rates [40].

Romania presents the follow-up data for the cohort at baseline, at six months, and at one year, with the exception of four CVRFs. This allows for the evolution of CVRFs to be assessed, with a considerable decrease at six months for all CVRFs for which data are available, and an increase at twelve months. In this study, after the initial assessment, cardiovascular prevention strategies were adopted. These included smokers in an anti-smoking programme, pharmacological treatment for those CVRFs that required it, and the promotion of lifestyle changes, which justifies the decrease in CVRFs at 6-month follow-up [42].

In Switzerland, comparing the cohorts of men of different ages, it was found that sedentary lifestyle, smoking, hypercholesterolaemia, and hypertriglyceridaemia were less prevalent in the older age group, but CVRFs such as HBP, DM, overweight, obesity, and metabolic syndrome had a higher prevalence [47].

By comparing countries where the studies conducted included people of younger age groups, prevalence rates of the most common CVRFs were lower than in studies that included older population groups.

The study conducted in Cyprus, which associated depressive symptoms with the prevalence of CVRFs, is worth highlighting. It can be understood that patients who had symptoms of depression were more predisposed to suffer from CVRFs, and it was also observed in the participants of this study that those with depressive symptoms had a higher prevalence of HBP, DM, obesity, and hypercholesterolemia [27].

Following this study, other research has been published that support this association between CVRFs and depression, especially HBP and depression, where it can be observed that there is a diagnosis of depression prior to HBP [48,49].

### 4.2. Insights to Inform Public Health Policies and Strategies

The reviewed studies consistently demonstrate a clear trend: the prevalence of CVRFs increases with age. This positive association is evident in findings from Belgium, where the most represented age group was 30 to 49 years, and higher rates of CVRFs were observed among older age groups. Similarly, data from Estonia indicated that the risk of coronary heart disease exceeded 10% in 34.6% of men and 14.4% of women aged 20 to 65 years, suggesting that cumulative risk increases with age.

Further evidence comes from large-scale studies such as EUROASPIRE V, which included participants aged 18 to 80, allowing for a detailed analysis of prevalence across age groups. In Italy, both national and regional samples of individuals aged 18 to 84 showed that obesity, hypertension (HBP), and diabetes mellitus (DM) were significantly more prevalent among older adults, particularly in the southern regions of the country.

This age-related increase in CVRFs aligns with the known pathophysiological processes of aging, in which metabolic regulation, blood pressure control, and glycaemic balance progressively deteriorate, thereby enhancing cardiovascular risk

Gender differences in the prevalence of CVRFs are also prominently reflected in available data. For instance, in a study conducted in Estonia, the estimated 10-year coronary heart disease risk was greater than 10% in 34.6% of men compared to 14.4% of women [30]. A similar pattern was observed in Germany, where men exhibited higher rates of risky alcohol consumption, smoking, and abdominal obesity across most federal states [4].

However, not all disparities favour males. In some countries, women show higher rates of physical inactivity. For example, a study conducted in Cyprus among individuals aged 65 and older found a higher prevalence of both CVRFs and depression in women, who accounted for 54.66% of the sample [27]. These findings point not only to a greater disease burden among older women but also to potential additional vulnerabilities from a psychosocial perspective.

Interestingly, EUROASPIRE V reported that smoking was the only CVRF with a higher prevalence in men, while all other risk factors were more common in women [9]. This reversal in trends underscores the importance of developing sex-specific prevention strategies, as generalised approaches may not be equally effective for both men and women.

The comparison between northern and southern European countries reveals distinct patterns in the distribution of CVRFs. In general, northern countries—such as Sweden, Germany, and the Netherlands—show higher prevalence of lifestyle-related risk behaviours commonly associated with modern living, including tobacco use and excessive alcohol consumption. For instance, in Germany, smoking was more prevalent in Berlin (40.7% among men) and in Bremen (30.9% among women) [4], while in Sweden, significant rates of obesity and dyslipidaemia were reported among older men [47].

Additionally, findings from the Netherlands highlighted a high prevalence of risk factors such as elevated cholesterol and abdominal obesity, affecting both sexes but with a higher representation among women [38].

It is worth noting that these northern countries generally have more robust and universal health systems, which may facilitate better detection and reporting of CVRFs—though this does not necessarily translate into lower prevalence rates.

Southern European countries—such as Italy, Spain, Portugal, and Greece—exhibit cardiovascular risk patterns more frequently associated with metabolic conditions, including obesity and diabetes. In Italy, for instance, data indicate a higher prevalence of obesity in the southern regions (31.5% in men and 23.5% in women), as well as elevated rates of diabetes (21.5% and 12.6%, respectively) [35]. A similar trend is evident in Spain, where studies such as DARIOS and Di@bet.es have reported a higher prevalence of obesity, metabolic syndrome, and hypertension in southern autonomous communities like Andalusia [43,46]. In Greece, a review of studies conducted between 1994 and 2016 documented a progressive increase in several cardiovascular risk factors—including obesity, smoking, and dyslipidaemia —partly linked to the economic crisis and the gradual abandonment of the traditional Mediterranean diet [2]. These findings underscore the critical role of social determinants of health—such as socioeconomic status, education, and access to health resources—in shaping the distribution and emergence of cardiovascular risk factors across southern Europe.

Health systems based on the Beveridge model, such as those in Spain and Sweden, provide universal access to healthcare, with a strong emphasis on prevention and health promotion. In these countries, there is a significant prevalence of CVRFs, with notable gender differences—women tend to have higher rates of obesity, while men exhibit a greater prevalence of hypertension and smoking. Spain has implemented various programmes to reduce cardiovascular risks; however, regional disparities in disease prevalence persist.

In countries with health insurance-based models, such as Germany and France, there is a high prevalence of hypertension, obesity, and diabetes. These countries have adopted prevention-oriented policies, including public awareness campaigns focused on healthy diets, physical activity, and the reduction in alcohol and tobacco consumption. In the Netherlands, prevention efforts are also centred on cardiovascular health, with particular attention on cholesterol control and the promotion of physical activity.

In Italy, regional differences are pronounced, with a higher prevalence of diabetes and obesity in the southern regions. Health policies emphasize the promotion of the Mediterranean diet and physical activity. In Poland, rural areas face greater challenges related to access and health education. In Portugal, policies are geared toward fostering healthy habits among the older population.

### 4.3. Limitations

This review has several limitations that should be considered. Firstly, some studies were excluded due to language restrictions, which may have resulted in the omission of relevant articles meeting other inclusion criteria. Additionally, studies that were not peer-reviewed or did not meet the minimum quality standards based on critical appraisal were excluded to ensure the robustness of the findings. All included studies employed cross-sectional observational designs which, while useful for assessing health status in specific populations at a given time, do not allow for causal inferences and are limited by potential non-representativeness of samples. Furthermore, a high level of heterogeneity was observed across all meta-analyses, primarily attributable to the inclusion of studies from diverse countries with distinct sociodemographic and methodological characteristics. Variability in measurement instruments across studies may have also contributed to these inconsistencies. In addition, although this review acknowledges differences in the definitions of CVRFs used across studies, no formal harmonisation or stratified analyses were performed to account for these discrepancies, which may limit the comparability and validity of the pooled estimates.

Another important limitation is the absence of subgroup and sensitivity analyses. Although a random-effects model was used to account for heterogeneity, the limited availability and variability of detailed data —such as stratification by age, geographic region, or study quality— precluded the possibility of conducting these analyses. This constraint reduces the granularity and interpretability of the pooled prevalence estimates and underscores the need for future research to adopt more standardised and comprehensive reporting practices that facilitate subgroup exploration, data harmonisation, and robustness of assessments.

## 5. Conclusions

The prevalence of cardiovascular risk factors across the European Union shows significant variability, shaped by demographic variables such as age, sex, and the characteristics of specific population subgroups. Among the most widespread cardiovascular risk factors are overweight, hypercholesterolaemia, and high blood pressure, while diabetes mellitus presents a comparatively lower prevalence.

These risk factors, together with the ongoing demographic shift toward an ageing population and increased life expectancy, have contributed substantially to the rising burden of morbidity. As a result, there is a growing proportion of individuals affected by chronic diseases and multimorbidity, placing considerable strain on healthcare systems through increased service demand and escalating costs.

The observed regional disparities further underscore the importance of developing and implementing targeted public health policies and preventive strategies. These should be tailored to the specific epidemiological and demographic contexts of each country or region, with the ultimate goal of reducing the incidence of cardiovascular risk factors and mitigating their long-term consequences for the health of the population and healthcare sustainability.

## Figures and Tables

**Table 1:** Critical reading, level of evidence (LE), and degree of recommendation (DR).

Reference	Type of Study	Critical Reading	Synthesis of the Evidence ^3^ LE/DR
Cardiovascular disease in Greece; the latest evidence on risk factors [2]	Systematic review	8/10 ^2^	LE: 2++ DR: B
Regionale Unterschiede in der Prävalenz von kardiovaskulären Risikofaktoren bei Männern und Frauen in Deutschland [4]	Cross-sectional study	High quality ^1^	LE: 2++ DR: B
Lifestyle and impact on cardiovascular risk factor control in coronary patients across 27 countries: Results from the European Society of Cardiology ESC-EORP EUROASPIRE V registry [9]	Cross-sectional study	High quality ^1^ *p* < 0.001–0.0001	LE: 2++ DR: B
Impact on the Prevalence of Cardiovascular Risk Factors in Wallonia, Belgium: A Population-Based Study [25]	Cross-sectional study	High quality ^1^ *p* < 0.0001–0.46	LE: 2++ DR: B
Age trends in prevalence of cardiovascular risk factors in Roma minority population of Croatia [26]	Cross-sectional study	High quality ^1^ *p <* 0.001–0.98	LE: 2++ DR: B
Depressive symptomatology and the prevalence of cardiovascular risk factors among older men and women from Cyprus; the MEDIS (Mediterranean Islands Elderly) epidemiological study [27]	Cross-sectional study	High quality ^1^ *p <* 0.001–0.98	LE: 2++ DR: B
Prevalence of risk factors in cardiovascular diseases in selected population of the Czech Republic [28]	Cross-sectional study	High quality ^1^ *p* < 0.001	LE: 2++ DR: B
Analysis of incidence and prevalence of cardiovascular risk factors and evaluation of their control in epidemiological survey in the Czech Republic [29]	Cross-sectional study	High quality ^1^	LE: 2++ DR: B
Prevalence of cardiovascular disease risk factors in Tallinn, Estonia [30]	Cross-sectional study	High quality ^1^ *p* < 0.001–1	LE: 2++ DR: B
Changes over time in the prevalence and treatment of cardiovascular risk factors, and contributions to time trends in coronary mortality over 25 years in the Lille urban area (northern France) [31]	Cross-sectional study	High quality ^1^ *p* < 0.0001–0.95	LE: 2++ DR: B
Prevalence of stroke/cardiovascular risk factors in rural Hungary—A cross—sectional descriptive study [32]	Cross-sectional study	High quality ^1^ *p* < 0.0001–0.038	LE: 2++ DR: B
Cardiovascular risk factors and frailty in a cross-sectional study of older people: implications for prevention [33]	Cross-sectional study	High quality ^1^ *p* < 0.001	LE: 2++ DR: B
Prevalence of ‘borderline’ values of cardiovascular risk factors in the clinical practice of general medicine in Italy: results of the BORDERLINE study [34]	Cross-sectional study	High quality ^1^ *p* < 0.05	LE: 2++ DR: B
Evidence on the prevalence and geographic distribution of major cardiovascular risk factors in Italy [35]	Cross-sectional study	High quality ^1^ *p* < 0.001–0.488	LE: 2++ DR: B
Classical rather than genetic risk factors account for high cardiovascular disease prevalence in Lithuania: A cross-sectional population study [36]	Cross-sectional study	High quality ^1^ *p* < 0.001–1	LE: 2++ DR: B
Prevalence and related risk factors of chronic kidney disease among adults in Luxembourg: evidence from the observation of cardiovascular risk factors (ORISCAV-LUX) study [37]	Cross-sectional study	High quality ^1^ *p* < 0.001–0.01	LE: 2++ DR: B
A comparison of the prevalence and clustering of major cardiovascular risk factors in the Netherlands and China [38]	Cross-sectional study	High quality ^1^ *p* < 0.05	LE: 2++ DR: B
Porównanie czynników ryzyka chorób sercowo-naczyniowych w województwach północnej i południowej części Polski [39]	Cross-sectional study	High quality ^1^ *p* < 0.001–0.1	LE: 2++ DR: B
The Differences in the Prevalence of Cardiovascular Disease, Its Risk Factors, and Achievement of Therapeutic Goals among Urban and Rural Primary Care Patients in Poland: Results from the LIPIDOGRAM 2015 Study [40]	Cross-sectional study	High quality ^1^ *p* < 0.01–0.92	LE: 2++ DR: B
Prevalence and management of cardiovascular risk factors in Portuguese living in Portugal and Portuguese who migrated to Switzerland [41]	Cross-sectional study	High quality ^1^ *p* < 0.001–0.99	LE: 2++ DR: B
Prevalence and Risk Factors of Metabolic Syndrome: A Prospective Study on Cardiovascular Health [42]	Cross-sectional study	High quality ^1^ *p* < 0.0009–0.71	LE: 2++ DR: B
Tratamiento y control de los factores de riesgo según el riesgo coronario en la población española del estudio DARIOS [43]	Pooled analysis of 11 population-based studies (Cross-sectional study)	High quality ^1^ *p* < 0.01–0.024	LE: 2++ DR: B
Prevalence of obesity and associated cardiovascular risk: the DARIOS study [44]	Pooled analysis of 11 population-based studies (Cross-sectional study)	High quality ^1^ *p* < 0.00–0.05	LE: 2++ DR: B
Síndrome metabólico en España: prevalencia y riesgo coronario asociado a la definición armonizada y a la propuesta por la OMS. Estudio DARIOS [45]	Pooled analysis of 11 population-based studies (Cross-sectional study)	High quality ^1^ *p* < 0.001	LE: 2++ DR: B
Prevalence of Cardiovascular Risk Factors in Spain: A Systematic Review [46]	Systematic review	10/10 ^2^	LE: 2++ DR: B
Prevalence of cardiovascular risk factors and the metabolic syndrome in middle-aged men and women in Gothenburg, Sweden [47]	Cross-sectional study	High quality ^1^ *p* < 0.001	LE: 2++ DR: B

^1^ Berra et al. [23]; ^2^ CASPe [21,22]; ^3^ Scottish Intercollegiate Guidelines Network (SIGN) [24]; LE: level of evidence; DR: degree of recommendation.

**Table 2:** General characteristics of the findings per country.

Country		*N* ^1^	Age Range	Male Sex (%)	Female Sex (%)
EUROASPIRE		8261	18–80	74.3	25.7
Belgium		1017	20–69	49.9	50.1
Croatia		19,070 ^2^ 430 ^3^	18–65 ^2^ 18–84 ^3^	35.12 ^3^	64.88 ^3^
Cyprus		300	>65	45.34	54.66
Czech Republic		1051 ^4^ 1812 ^5^	>40 in males ^5^ >50 in females ^5^	45.86 ^4^ 53.03 ^5^	54.14 ^4^ 46.97 ^5^
Estonia		1111	20–65	46	54
France		860 ^6^ 1021 ^7^ 1021 ^8^ 1636 ^9^	40–64 ^6–9^	52.7 ^6^ 49 ^7^ 50.4 ^8^ 47.3 ^9^	47.3 ^6^ 51 ^7^ 49.6 ^8^ 52.7 ^9^
Germany		62,606	18–99 in males 18–100 in females	48.9	50.9
Greece	EPIC STUDY	26,913	25–86		
	NAOUSSA	1937	15–73		
	MEDICAL EXPRESS	2805	20–94		
	ATTICA	3042	18–89		
	HYPERTENSHELL	11,540	>17		
	National Epidemiological Survey	17,887	20–70		
	A Nutrition & Health Survey	5003	18–74		
	ATTICA 1	2101	>22		
	Hellas Health I	1005	>18		
	Panagiotakos et al. [27]	10,141	18–95		
	Hellas Health II	1490	>18		
	Nationwide survey	3007	>18		
	Hellenic Statistical Authority health survey for 2009	6325	>15		
	ATTICA 2	2583	>17		
	Hellas Health IV	1008	>18		
	HYDRIA	4011	>18		
	GAST Study	4359	>15		
	HNNHS	4574	No age range is specified		
	SARONIKOS	2636	20–65		
	Hellenic Statistical Authority health survey for 2014	8223	>15		
	Hellas Health V	1001	>15		
Hungary		546	14–83		
Ireland		4330	55–67	44.5	55.5
Italy		692 ^10^ 24,213 ^11^	>18 ^11^	54.5 ^10^ 52 ^11^	45.5 ^10^ 48 ^11^
Lithuania		435	40–60	49.7	50.3
Luxembourg		1270 1331 ^12^	18–69	48.7	51.3
Netherlands		6542	>18	44.8	55.2
Poland		7376 ^13^	40–65 ^13^		
		13,724 ^14^	No age range is specified ^14^	36.7 ^14^	63.3 ^14^
Portugal		1550	35–65	37.3	62.7
Romania		117	30–79	45.3	54.7
Spain		27,903 ^15^ 28,887 ^16^ 24,670 ^17^	35–74 ^15–17^	46 ^15^ 46.5 ^16^	54 ^15^ 53.5 ^16^
Sweden		1927	Males born in 1953 and 1943 and females born in 1953	30.9 ^18^ 34.5 ^19^	34.6 ^18^

^1^ Sample size; ^2^ Croatian sample; ^3^ Roma sample; ^4^ Pavlík et al. [28]; ^5^ Chmelík Z et al. [29]; ^6^ MONICA 1986–1988 [31]; ^7^ MONICA 1995–1996 [31]; ^8^ MONALISA [31]; ^9^ ELISABET [31]; ^10^ BORDERLINE study [34]; ^11^ National Health Survey [35]; ^12^ sample for diabetes mellitus only; ^13^ Trzeciak et al. [39]; ^14^ Studziński et al. [40]; ^15^ Baena-Díez et al. [43]; ^16^ Félix Redondo et al. [44]; ^17^ Fernández-Bergés et al. [45]; ^18^ born in 1953; ^19^ born in 1943.

**Table 3:** Prevalence of CVRFs by country and study.

Country		*N*	High Blood Pressure (%)	Diabetes Mellitus (%)	Dyslipidaemia (%)	Sedentary Lifestyle (%)	Metabolic Syndrome (%)	Overweight (%)	Obesity (%)	Smoking Habit (%)	Hypercholesterolemia (%)	Hypertriglyceridemia (%)	Alcohol Use (%)	Fruit and Vegetables Intake (%) ^12^	Population Group
EUROASPIRE		8261	**42**	29		**42**			38/59 ^1^	**19**	---	---	---	---	Total
			**42**	28.3		39.3			34.8/51.9 ^1^	**20.9**	---	---	---	---	Males
			42.4	33.4		**48.6**			45.9/77.8 ^1^	**12.9**	---	---	---	---	Females
Belgium		1017	31.2	**6.52**	**65.7**	52.2	---	---	---	25	---	---	---	---	Total
			---	---	---	---	---	---	---	---	---	---	---	---	Males
			---	---	---	---	---	---	---	---	---	---	---	---	Females
Croatia (Croatian sample)		19,070	---	---	---	---	---	---	---	---	---	---	---	---	Total
			40.7	---	---	---	---	42	**19.8**	34.1	**72**	49.2	---	---	Males
			33.1	---	---	---	---	30.8	**17.8**	26.6	**67.5**	26.1	---	---	Females
Croatia (Roma sample)		430	26.3	---	---	---	---	28.1	**21**	**70.2**	67.7	43	---	---	Total
			26	---	---	---	---	33.6	**18.1**	**71.5**	64.4	45.6	---	---	Males
			26.4	---	---	---	---	25.2	**22.6**	**69.4**	69.5	41.6	---	---	Females
Cyprus		300	59.7	18	---	**62.3**	---	---	31.7	**7**	49.3	---	9	---	Total
			**60**	18	---	49	---	---	24	**14**	36	---	15	---	Males
			59	18	---	**73**	---	---	38	**1**	55	---	4	---	Females
Czech Rep. [28]		1051	---	**15.6**	---	---	---	36.8	18.1	---	**50.8**	20	---	---	Total
			---	**18.9**	---	---	---	48.8	20.3	---	**52.9**	24.9	---	---	Males
			---	**12.8**	---	---	---	26.7	16.3	---	**49**	15.8	---	---	Females
Czech Rep. [29]		1812	**50.6**	**14**	---	---	---	40.6	32	29.4	---	---	---	---	Total
			**51**	**14**	---	---	---	49	32	36	---	---	---	---	Males
			**50**	**14**	----	---	---	31	32	22	---	---	---	---	Females
Estonia		1111	31.1	**3.3**	---	---	22.9	55	46.3 ^1^	27.8	**62.3**	23.5	---	---	Total
			36.8	**4**	---	---	29.1	66.2	48.5 ^1^	36.1	**63.8**	30.9	---	---	Males
			26.1	**2.7**	---	---	17.5	48.3	44.5 ^1^	20.5	**60.9**	17	---	---	Females
France MONICA 86–88		860	63.8	---	**66.6**	---	---	55 ^2^	---	**31.2**	---	---	---	---	Total
			70.5	---	**71.1**	---	---	59.6 ^2^	---	**44.1**	---	---	---	---	Males
			56.6	---	**60.9**	---	---	49.8 ^2^	---	**16.7**	---	---	---	---	Females
France MONICA 95–96		1021	45.8	**9.2**	57.2	---	---	**57.7** ^2^	---	24.2	---	---	---	---	Total
			49.5	**9.5**	**66.2**	---	---	62.5 ^2^	---	33.1	---	---	---	---	Males
			42.4	**8.9**	48.5	---	---	**53** ^2^	---	15.5	---	---	---	---	Females
France MONALISA		1021	44.8	**6**	53	---	---	**57.5** ^2^	---	20.4	---	---	---	---	Total
			48	**7.8**	60.8	---	---	**64.5** ^2^	---	22.4	---	---	---	---	Males
			45	**4.1**	45	---	---	**50.4** ^2^	---	18.4	---	---	---	---	Females
France ELISABET		1636	38.2	**7.6**	49.9	---	---	**57** ^2^	---	20.8	---	---	---	---	Total
			42.5	**10.1**	58.3	---	---	**65.1** ^2^	---	24.7	---	---	---	---	Males
			34.3	**5.4**	42.2	---	---	**49.8** ^2^	---	17.3	---	---	---	---	Females
Germany ^3^		62,606	31.7	**9**	29.4	**35.3**	---	---	16	28.3	---	---	26.7	19.8	Total
			32.5	**8.7**	31	**35.4**	---	---	16.3	32.6	---	---	32.8	32.6	Males
			31.1	**9.2**	28.2	**35.3**	---	---	15.7	24.9	---	---	21.7	24.9	Females
Greece ^4^	EPIC Study	26,913	44.4	---	---	---	---	---	---	---	---	---	---	---	Total
	NAOUSSA	1937	30.5	---	---	---	---	---	---	---	---	---	---	---	Total
	MEDICAL EXPRESS	2805	20.1	8.7	---	---	---	---	18.4	---	---	---	---	---	Total
	ATTICA	3042	30	7	---	---	---	---	18	43	39	---	---	---	Total
	HYPERTENSHELL	11,540	31.1	---	---	---	---	---	25.5	---	---	---	---	---	Total
	National Epidemiological Survey	17,887	**8.6**	4.2	---	---	---	---	22.3	---	13.3	---	---	---	Total
	A Nutrition & Health Survey	5003	15.6	6	---	---	---	---	13.3	41.5	19.2	---	---	---	Total
	ATTICA 1	2101	40	12	---	---	---	---	20	38	54	---	---	---	Total
	Hellas Health I	4005	---	---	---	---	---	---	18	**43.1**	---	---	---	---	Total
	Panagiotakos et al. [27]	10,141	14.9	5.7	---	---	---	---	**11.8**	37.1	**10.6**	---	---	---	Total
	Hellas Health II	1490	---	---	---	---	---	---	18.9	42.6	---	---	---	---	Total
	Nationwide survey	3007	17.7	6.5	---	---	---	---	16.4	36.1	14	---	---	---	Total
	Hellenic Statistical Authority health survey for 2009	6325	20.2	7.9	---	---	---	---	17.3	37.9	15	---	---	---	Total
	ATTICA 2	2583	**52**	**20**	---	---	---	---	30	33	**62**	---	---	---	Total
	Hellas Health IV	1008	---	---	---	---	---	---	18.2	38.1	---	---	---	---	Total
	HYDRIA	4011	41.7	11.4	---	---	---	---	**34.9**	35.7	13.4	---	---	---	Total
	GAST Study	4359	---	---	---	---	---	---		38.2	---	---	---	---	Total
	HNNHS	4574	13.3	**3.6**	---	---	---	---	15.5	33.8	16.7	---	---	---	Total
	SARONIKOS	2636	27.2	11.1	---	---	---	---		38.9	23.8	---	---	---	Total
	Hellenic Statistical Authority health survey for 2014	8223	20.9	9.2	---	---	---	---	17	**32.6**	15.4	---	---	---	Total
	Hellas Health V	1001	15	6	---	---	---	---	17	37	12	---	---	---	Total
Hungary			---	---	---	---	---	---	---	---	---	---	---	---	Total
			23	**4.6**	---	---	---	**60**	---	39	26.9	36.5	12.7	---	Males
			32.7	**4.4**	---	---	---	**66.5**	---	22	30.5	12.2	0.84	---	Females
Ireland		4330	83.2	**5.7**	---	54.4	---	**73.5** ^2^	---	17.1	57.8	---	---	---	Total
			---	---	---	---	---	---	---	---	---	---	---	---	Males
			---	---	---	---	---	---	---	---	---	---	---	---	Females
Italy (BORDERLINE study)		692	**59.4**	18.6	---	---	---	---	**16.9**	24.4	43.8	---	---	---	Total
			**66.7**	22.8	---	---	---	---	**16.5**	30.8	46	---	---	---	Males
			**55.1**	**15.1**	---	---	---	---	17.2	19.1	41.9	---	---	---	Females
Italy (National Survey)		24,213	**59.6**	**15.3**	59	---	---	24.7	22.6/56.7 ^1^	19.8	29.6	45.5	---	---	Total
			**70**	**19**	60.3	---	---	56.7	26.1/57 ^1^	25.2	26.8	52	---	---	Males
			48.3	**11.2**	**55.7**	---	---	31.6	18.9/56.4 ^1^	14	32.7	48.1	---	---	Females
Lithuania		435	**72.7**	---	---	32.9	---	---	---	**21**	---	---	84	---	Total
			---	---	---	---	---	---	---	---	---	---	---	---	Males
			---	---	---	---	---	---	---	---	---	---	---	---	Females
Luxembourg		1270 (1331 for diabetes)	35.7	**3.7**	---	---	---	---	22	22	---		**85.7**	---	Total
			---	---	---	---	---	---	---	---	---	---	---	---	Males
			---	---	---	---	---	---	---	---	---	---	---	---	Females
Netherlands		6542	22	**2.9**	---	---	---	36.3	12.6	23	**48.4**	---	19	---	Total
			29.9	**3.6**	---	---	---	44.3	11.4	25.7	**50.4**	---	28.3	---	Males
			15.6	**2.3**	---	---	---	29.8	13.6	20.9	**46.8**	---	11.6	---	Females
Poland ^5^		7376	37	**8.5**	---	---	---	---	---	29	**64.7**	---	---	---	Total
			33.8	**10**	---	---	---	---	---	29.5	**61.2**	---	---	---	Norte
			42.1	**6.2**	---	---	---	---	---	28.1	**70.2**	---	---	---	Sur
Poland ^6^		6696	49.37	**13.13**	**85.22**	---	---	38.75	37.53/46.40 ^1^	15.77	---	---	---	---	Total rural
		7028	49.54	**13.72**	**84.22**	---	---	40.15	32.34/43.18 ^1^	17.74	---	---	---	---	Total urban
		4195	47.44	**11.61**	**82.5**	---	---	36.42	35.69/56.16 ^1^	13.13	---	---	---	---	Rural females
		4495	47.88	**11.88**	**82.27**	---	---	36.11	30.37/50.99 ^1^	16.64	---	---	---	---	Urban females
		2501	52.62	**15.67**	**89.76**	---	---	42.66	40.62/30.03 ^1^	20.19	---	---	---	---	Rural males
		2533	52.61	**16.98**	**87.68**	---	---	47.34	35.85/29.33 ^1^	19.70	---	---	---	---	Urban males
Portugal		1550	39.6	**6**	---	---	---	---	23.4/30.6 ^1^	25.5	72.2	---	---	---	Total
			---		---	---	---	---	---	---	---	---	---	---	Males
			---		---	---	---	---	---	---	---	---	---	---	Females
Spain		27,903	29	**14**	34	---	31.7 ^7^	**42.6 ^8^**	28.2^8^	---	---	---	---	---	Total
			---	**---**	---	---	32 ^7^	**50.7 ^8^**	**28 ^8^**	---	---	---	---	---	Males
			---	**---**	---	---	29 ^7^	**35.6 ^8^**	**28.3 ^8^**	---	---	---	---	---	Females
Romania		117	70.08	**33.33**	---	---	68.37	---	**76.92**	---	---	61.53	---	---	At the start
			41.02	**24.7**	---	**61.52**	59	---	52.1	54.45	---	34.18	37.6	36.76	At 6 months
			43.58	**25.6**	---	---	**63.2**	---	54.7	---	---	35.8	---	---	At 12 months
Sweden			46.2	**4**	---	17.7	16.1 ^9^ 22.6 ^10^ 26 ^11^	**63.4**	15.3	21.5	32.4	38.2	---	---	Males *n* = 53
			36	**2**	---	13.8	10.5 ^9^ 12.6 ^10^ 15.8 ^11^	**45.9**	15.1	26.1	22.6	17.3	---	---	Females *n* = 53
			66.7	**6.4**	---	11.6	19.9 ^9^ 26.1 ^10^ 35 ^11^	**71.6**	16.6	15.1	26	22.4	---	---	Males *n* = 43

^1^ Data on abdominal obesity; ^2^ data on overweight or obesity; ^3^ the exposure population is the participants of the GEDA survey, and for each CVRF the sample differs; ^4^ the minimum and maximum prevalence values of a total of 22 references are presented; ^5^ Trzeciak et al. [39]; ^6^ Studziński et al. [40]; ^7^ Fernández-Bergés et al. [45]; ^8^ Félix-Redondo et al. [44]; ^9^ National Cholesterol Education Program (NCEP); ^10^ American Heart Association (AHA); ^11^ International Diabetes Federation (IDF); ^12^ low fruit and vegetable intake; -- data not present; In bold, the most prevalent CVRF in each study is presented by sex, while the least prevalent CVRF is shown in regular font. For Greece, the most and least prevalent CVRFs are presented in bold and regular font, respectively, for each CVRF.

## Data Availability

All data are available within this article. The protocol of this systematic review has been registered (https://doi.org/10.17605/OSF.IO/Z86XD).

## Abbreviations

The following abbreviations are used in this manuscript:

CVRF	Cardiovascular Risk Factor
CVD	Cardiovascular Disease
HBP	High Blood Pressure
DM/DM2	Diabetes Mellitus/Type 2 Diabetes Mellitus
MS	Metabolic Syndrome
Hcol	Hypercholesterolemia
Htri	Hypertriglyceridemia
SCORE	Systematic Coronary Risk Evaluation
REGICOR	Registre Gironí del Cor
LE	Level of Evidence
DR	Degree of Recommendation
SING/SIGN	Scottish Intercollegiate Guidelines Network
CASPe	Critical Appraisal Skills Programme español
IDF	International Diabetes Federation
AHA	American Heart Association
NCEP	National Cholesterol Education Program
EU	European Union
PRISMA	Preferred Reporting Items for Systematic Reviews and Meta-Analyses
CoCoPop	Condition, Context, Population
VHL	Virtual Health Library
LILACS	Latin American and Caribbean Health Sciences Literature
Redalyc	Red de Revistas Científicas de Acceso Abierto Diamante

## Appendix A. Supplementary Tables

**Table A1:** Search strategy.

Database	Date	Search Equations	Total Articles	Selected Articles (Titles And Abstracts)	Selected Articles (After Full Reading)	Selected Articles (After Removing Duplicates)
Pubmed	18 June 2024	Cardiovascular risk factors AND prevalence AND Europe	621	54	3	1
Virtual Health Library	18 June 2024	Cardiovascular risk factors AND prevalence AND Europe	130	12	2	0
LILACS	26 March 2025	Cardiovascular risk factors AND prevalence AND Europe	2	0	0	0
Dialnet	26 March 2025	Cardiovascular risk factors AND prevalence AND Europe	1	0	0	0
Scielo	26 March 2025	Cardiovascular risk factors AND prevalence AND Europe	6	0	0	0
Redalyc	26 March 2025	Cardiovascular risk factors AND prevalence AND Europe	2418	23	23	0
Pubmed	18 June 2024	Cardiovascular risk factors AND prevalence AND Germany	550	32	1	1
Pubmed	18 June 2024	Cardiovascular risk factors AND prevalence AND Belgium	161	28	1	1
Pubmed	18 June 2024	Cardiovascular risk factors AND prevalence AND Croatia	241	14	4	1
Pubmed	18 June 2024	Cardiovascular risk factors AND prevalence AND Denmark	238	0	0	0
Pubmed	18 June 2024	Cardiovascular risk factors AND prevalence AND Ireland	108	1	1	1
Pubmed	18 June 2024	Cardiovascular risk factors AND prevalence AND Latvia	9	0	0	0
Pubmed	18 June 2024	Cardiovascular risk factors AND prevalence AND Luxembourg	19	1	1	1
Pubmed	18 June 2024	Cardiovascular risk factors AND prevalence AND Netherlands	524	1	1	1
Pubmed	18 June 2024	Cardiovascular risk factors AND prevalence AND Sweden	359	27	1	1
Pubmed	18 June 2024	Cardiovascular risk factors AND prevalence AND Italy	635	5	5	2
Pubmed	18 June 2024	Cardiovascular risk factors AND prevalence AND Bulgaria	21	0	0	0
Pubmed	18 June 2024	Cardiovascular risk factors AND prevalence AND Slovakia	26	3	0	0
Pubmed	18 June 2024	Cardiovascular risk factors AND prevalence AND Estonia	22	1	1	1
Pubmed	18 June 2024	Cardiovascular risk factors AND prevalence AND Greece	7	4	4	1
Pubmed	18 June 2024	Cardiovascular risk factors AND prevalence AND Malta	6	0	0	0
Pubmed	18 June 2024	Cardiovascular risk factors AND prevalence AND Poland	178	3	2	2
Pubmed	18 June 2024	Cardiovascular risk factors AND prevalence AND Czech Republic	59	2	2	2
Pubmed	18 June 2024	Cardiovascular risk factors AND prevalence AND Austria	130	0	0	0
Pubmed	18 June 2024	Cardiovascular risk factors AND prevalence AND Cyprus	27	2	2	1
Pubmed	18 June 2024	Cardiovascular risk factors AND prevalence AND Slovenia	32	1	0	0
Pubmed	18 June 2024	Cardiovascular risk factors AND prevalence AND Finland	143	0	0	0
Pubmed	18 June 2024	Cardiovascular risk factors AND prevalence AND Hungary	39	2	2	1
Pubmed	18 June 2024	Cardiovascular risk factors AND prevalence AND Lithuania	12	1	1	1
Pubmed	18 June 2024	Cardiovascular risk factors AND prevalence AND Portugal	77	2	2	1
Pubmed	18 June 2024	Cardiovascular risk factors AND prevalence AND Romania	69	2	1	1
Pubmed	18 June 2024	Cardiovascular risk factors AND prevalence AND Spain	233	4	4	4
Pubmed	18 June 2024	Cardiovascular risk factors AND prevalence AND France	215	1	1	1
Total			7318	218	55	26
